# Influence of Nutrients on Kidney Diseases

**DOI:** 10.3390/nu14061234

**Published:** 2022-03-15

**Authors:** Yoshiyuki Morishita, Naoki Nakagawa

**Affiliations:** 1 Division of Nephrology, Department of Integrated Medicine, Saitama Medical Center, Jichi Medical University, 1-847, Amanuma, Omiya-ku, Saitama 330-8503, Japan; 2 Division of Cardiology, Nephrology, Pulmonology and Neurology, Department of Internal Medicine, Asahikawa Medical University, 2-1-1-1 Midorigaoka Higashi, Asahikawa 078-8510, Japan; naka-nao@asahikawa-med.ac.jp

Kidneys filter and reabsorb various nutrients and electrolytes [1]. They also synthesize many hormones, such as renin, erythropoietin, and prostaglandins, to maintain homeostasis [1]. Kidney diseases, such as chronic kidney disease (CKD) and acute kidney injury (AKI), can cause many types of nutritional abnormality secondary to various pathologies, and these can result in malnutrition, anemia, and mineral bone disorder, among other disorders [1].

The prevalence of CKD is increasing worldwide, and this is accompanied by a substantial socioeconomic burden [2]. CKD is progressive; many patients experience a deterioration in renal function and require renal replacement therapy [3]. CKD is also associated with a higher risk of developing cardiovascular disease [4]. Studies of salt and protein restriction have been conducted to evaluate their renoprotective effects in patients with CKD [5,6]. However, dietary restriction may contribute to sarcopenia and frailty, particularly in older individuals [7]. Therefore, the relationships among nutritional status, quality of life, and prognosis in patients with CKD require further investigation.

AKI, which refers to acute kidney tissue damage, is characterized by an increase in serum creatinine concentration and lower urine output [8]. AKI is caused by various pathological conditions, such as lower renal blood flow; dehydration; sepsis; heart failure; surgical treatment; and acute kidney parenchymal damage, caused by various kidney diseases, drugs, or urinary retention, owing to urinary tract tumor or stones [8]. The nutritional support required for patients with AKI depends upon the pathology, catabolic rate, and presence or absence of acute and chronic comorbidities [9].

Nutrients and kidney diseases are closely related. Therefore, this Special Issue focuses on the influence of nutrients on kidney diseases, with the intention of updating knowledge regarding the role of nutrients in the pathogenesis, molecular mechanisms, clinical study, diagnosis, and treatment of kidney diseases.

Meir at al. conducted a cross-sectional study of the methods that could be used to estimate the amount of inorganic phosphate (P) ingested, which is preferable to the measurement of the amount of P-containing proteins ingested, in 71 patients with stage 2 or 3 CKD and normal serum P concentration [10]. They found that the amount of inorganic P ingested was associated with the ratio of urinary P to urea nitrogen excreted (P/UUN ratio), but that the amount of P ingested from animal and vegetable sources was not associated with the P/UUN ratio in 24 h urine samples [10]. Thus, the P/UUN ratio in 24 h urine samples may be a useful means of estimating inorganic P intake [11]. This finding may help in the management of mineral bone disorder in patients with CKD, in which the estimation and control of serum P concentration are important.

Perez-Torres et al. demonstrated the long-term (2-year) effects of a nutritional intervention, referred to as a nutritional educational program, in 186 patients with advanced CKD [11]. This nutritional educational program consisted of an individualized diet plan based on each patient’s initial nutritional status, four nutritional education sessions, a nutritional assessment, and 6 months of monitoring [11]. The intervention was administered by a single dietitian and aimed to provide a personalized dietary prescription, including the provision of 25–35 kcal/kg/day energy and 0.75–1.0 g/kg/day protein, according to the Kidney Disease Outcomes Quality Initiative (K/DOQI) guidelines [11]. The authors assessed the patients’ protein and energy intakes, the phosphorus and potassium contents of the foods, the cooking techniques used, and a fourth parameter selected according to the patients’ specific needs [11]. They developed a detailed dietary plan after obtaining information from a 3-day dietary record [11], and used photographic albums to estimate portion sizes and explain to patients how to read and understand food labels [11]. They found that this problem significantly reduced the prevalence of hospitalization in those who underwent the intervention (13.7%) versus those who did not (26.7%) [11]. However, there was no clear independent association between the mortality rate and participation in the nutritional educational problem (*p* = 0.05) [11]. Thus, the study showed that participation in an individualized nutritional education reduces the risk of hospital admission, which may imply that it helps to maintain quality of life in patients with advanced CKD.

Mika et al. characterized the fatty acid (FA) profiles that might contribute to the development of cardiovascular diseases using gas chromatography–mass spectrometry in 198 renal transplant recipients 12 months postoperatively, and compared the results with those of 48 healthy individuals [12]. They found that the main differences between the renal transplant recipients and controls were in the contents of branched-chain FAs, monounsaturated FAs, and n-6 polyunsaturated FAs (PUFAs) [12]. They also found that the abnormalities in the FA profiles of the renal transplant recipients tended to gradually normalize postoperatively [12]. In addition, they found a close inverse relationship between n-3 PUFA content and the presence of inflammation [12]. The most profound alterations in the FA profile were identified in patients with impaired graft function (glomerular filtration rate < 45 mL/min) [12]. These findings suggest that FA supplementation and/or nutritional education are needed for patients who undergo renal transplantation to improve their prognosis. For this reason, it is important to conduct a clinical study to determine whether FA supplementation and/or nutritional education reduce the incidence of cardiovascular disease and help maintain the function of transplanted kidneys.

Park et al. investigated the causal effects of homocysteine and vitamin Bs in the homocysteine metabolic pathway, including folate (vitamin B9) and cobalamin (vitamin B12), on the estimated glomerular filtration rate (eGFR) by Mendelian randomization analysis using two largest genotyped datasets of 567,460 European ancestry individuals for kidney function traits [13]. They found that high genetically predicted blood homocysteine levels were significantly associated with low eGFR [13]. They also found that genetically predicted high blood folate (vitamin B9) levels were significantly associated with high eGFR [13]. However, those of cobalamin (vitamin B12) were not significantly correlated eGFR [13]. These finding suggests that clinical studies are needed to determine how interventions aimed at reducing homocysteine may reduce renal impairment in patients with CKD [13].

Champan et al. investigated the effects of acute beetroot juice ingestion on the renal hemodynamics of 14 healthy young adults during normoxia and mild hypercapnia [14]. They measured blood-flow velocity and vascular resistance in the renal and segmental arteries using Doppler ultrasonography during 5 min of breathing a carbon dioxide (CO_2_) gas mixture consisting of 3% CO_2_, 21% O_2_, and 76% N_2_ before and 3 h after the consumption of 500 mL of beetroot juice [14]. They found that acute beetroot juice ingestion does not attenuate reductions in renal perfusion, including blood flow velocity and vascular resistance in the renal and segmental arteries in acute mild hypercapnia in healthy young adults following beetroot juice injection [14] These results suggest beetroot juice injection does not alter renal hemodynamics during normoxia and mild hypercapnia, at least in healthy young adults. These results might be worth investigating in patients with CKD.

Martínez-Pineda et al. investigated whether potassium additives in processed foods might represent a hidden risk for excessive potassium intake in patients with CKD [15], listed the European Union-authorized food additives that contain potassium and their conditions of use, and classified them according to their risk for patients with CKD [15]. In addition, they measured the frequency of inclusion of potassium-containing additives in processed foods in 715 European products [15], finding that 37.6% contained at least one potassium-containing additive [15]. The food categories that showed the highest prevalence of the addition of potassium were bread products, meat derivatives, non-alcoholic beverages, ready-to-eat products, and cereal derivatives [15]. These findings imply that careful evaluation of foodstuffs is necessary to identify hidden potassium to ensure appropriate nutritional management of patients with CKD who are at risk of hyperkalemia.

Maraj et al. investigated the relationships of α1-acid glycoprotein (AGP), an acute-phase protein, and other laboratory parameters with nutrient intake in 59 patients undergoing maintenance hemodialysis [16]. They found that the serum concentration of AGP negatively correlated with the dietary intakes of plant protein, potassium, copper, vitamin B6, and folate, but not with those of animal or total protein [16]; and also that serum AGP was independently positively associated with serum C-reactive protein concentration [0.46 ± 0.13 (β ± SE), *p* = 0.001] [16]. These findings suggests that nutrition and inflammation are related in such patients.

Krisgher et al. investigated the relationship between kidney function, anemia, and diabetes mellitus in 203 male Guatemalan sugarcane workers [17]. They found that the renal function (eGFR 18 mL/min/1.73 m^2^) of those who were anemic (Hg < 13 g/dL) was poorer than in those who were not, and was also poorer (eGFR 8 mL/min/1.73 m^2^) in those who had high glycosylated hemoglobin values (HbA1c ≥ 5.7%; *p* < 0.01) [17]. These results may suggest that anemia, glucose metabolism, and renal function interact.

The importance of assessing nutritional status in patients with CKD has been discussed in two previous reviews. Nakagawa et al. summarized the available evidence regarding the relationships of geriatric nutritional risk index, which is calculated using body mass, height, and serum albumin concentration, with all-cause and cardiovascular mortality in patients with CKD [18]; and Nakashima et al. discussed the mechanisms whereby insulin resistance plays pivotal roles in the development of cardiovascular disease in patients with CKD, and the evidence regarding insulin resistance in this population [19]. These reviews help readers understand the factors which may be related to the development of cardiovascular disease in patients with CKD.

In conclusion, interdisciplinary studies involving clinical, geriatric, and other related components, as in this Special Issue, are likely to substantially aid our understanding of the influences of nutrients on kidney diseases. However, further research is required to improve knowledge regarding these relationships.
